# Field Monitoring of *Drosophila suzukii* and Associated Communities in South Eastern France as a Pre-Requisite for Classical Biological Control

**DOI:** 10.3390/insects8040124

**Published:** 2017-11-16

**Authors:** Laurent Kremmer, Marcel Thaon, Nicolas Borowiec, Jean David, Marylène Poirié, Jean-Luc Gatti, Nicolas Ris

**Affiliations:** 1 “Institut Sophia Agrobiotech”, INRA, CNRS, Université Côte d’Azur, 400 route des Chappes—BP 167, 06903 Sophia-Antipolis, France; laurent.kremmer@inserm.fr (L.K.); marcel.thaon@inra.fr (M.T.); nicolas.borowiec@inra.fr (N.B.); Marylene.POIRIE@unice.fr (M.P.); jean-luc.gatti@inra.fr (J.-L.G.); 2 Laboratoire Évolution, Génomes, Comportement, Écologie (EGCE), CNRS, IRD, Université Paris-sud, Université Paris-Saclay, CNRS - Bât. 13, 91198 Gif sur Yvette CEDEX, France; Jean.David@egce.cnrs-gif.fr

**Keywords:** spotted wing Drosophila, biological control, risk assessment, drosophilids survey, parasitoids

## Abstract

The spotted wing Drosophila, *Drosophila suzukii* (Ds), became a major economic pest for fruit production since its establishment in Europe and America. Among potential control methods, only classical biological control appears to be a mean of sustainably regulating Ds in both cultivated and natural habitats. In the frame of risk assessment, pre-release surveys were carried out in a restricted but highly heterogeneous area in the south-east of France using traps and deliberate field exposures of Ds and *D. melanogaster* larvae/pupae. Although Ds abundance varied according to sampling methods, it was found to be pervasive and to produce offspring and adults in most conditions (spatial and seasonal). Its main limits are some specific abiotic conditions (i.e., desiccation) as well as interspecific competition. Indeed, Ds mostly co-occurred with *D. busckii* and *D. hydei*, probably due to common phenology and/or ecological requirements. These two species thus deserve more attention for risk assessment. The main indigenous parasitoids collected belonged to two pupal species, *Trichopria cf drosophilae* and *Pachycrepoideus vindemmiae*, but their presence was observed late in the autumn and mainly in cultivated areas. Results are discussed in a comparison of the methodological approaches for monitoring Drosophilids and the benefits-risks assessment of classical biological control.

## 1. Introduction

Over the past decade, the spotted wing Drosophila, *Drosophila suzukii* Matsumura 1931 (Ds) (Diptera: Drosophilidae), has spread worldwide and becomes a major economic problem for many fruits productions (stone fruits and berries), especially in Europe and the United States [1,2,3,4]. In response, various methods of pest control have been deployed, including prophylaxis, (bio)chemical control, mass trapping, or the use of expensive protective nets [1,5,6,7,8,9]. However, current Integrated Pest Management (IPM) strategies appear insufficient or too costly for the most affected crops, and the use of pesticides is problematic because of their toxicity or the residual contamination of the fruits after pre-harvest treatments. Among other pest control methods, classical biological control—defined as the deliberate introduction of an exotic biological control agent for its perennial establishment and long-term control of the target pest [10]—is a possible approach for regulating Ds, not only in cultivated areas but also in more natural habitats that can serve as refuges and food sources. This latter aspect of the management of Ds is particularly relevant insofar as Ds is known to be highly polyphagous in invaded areas [11,12,13].

The deliberate introduction of an exotic biological control agent in the frame of classical biological control often requires a formal agreement [14,15,16]. In France, this agreement is issued by both the Ministry of Agriculture and the Ministry of Environment after an independent investigation led by the ANSES Safety Agency to weigh the (possible) benefits and drawbacks of the introduction (“Arrêté du 28 June 2012—AGRG1225395A”). Applicants must, notably, conduct a reliable “pre-release” local survey to (i) assess potential spatiotemporal variations of the target pest populations, (ii) assess the presence of native natural enemies, and (iii) identify the main non-target species that could potentially be affected.

In this context, a multi-site survey was carried out in the south-east of France. This geographical area was selected for reasons of convenience, due to the previous successful establishment of exotic species since 2010 (see for example [17]), but above all for its particularly marked environmental heterogeneity (climates, habitats, host plants) which encompasses several conditions found in France and neighboring countries.

To this end, we used two types of standardized traps, combined with deliberate field exposures of Ds or *Drosophila melanogaster* larvae, to (i) better document the biology of Ds in this area, (ii) identify ecologically close drosophilids and their potential interspecific interactions, and (iii) evaluate the role of natural enemies with emphasis on *Drosophila* parasitoids. All the results are discussed in relation to the available literature (laboratory and field) with an emphasis on the European context.

## 2. Materials and Methods

### 2.1. Sampling Location

The survey was carried out in south-eastern France (“Alpes-Maritimes”), near the Italian border, on six selected sites: Biot (BIO), Cipières (CIP), Col Ferrier (FER), La Baronne (BAR), Saint Jeannet (STJ), Tignet (TIG) (see Table 1 and Supplementary Material Figure S1). Although the surface is rather small (about 400 km^2^), the selected areas have marked climatic, geographical, and ecological heterogeneity. There is, notably, a strong altitudinal gradient between the seaside/the lower part of the Var valley and the mountainous regions of the backland. Three bioclimatic zones can thus be defined: Thermo- (limited to the very near, urbanized neighborhood of the seafront), Meso- (BAR, BIO, STJ, and TIG) and Supra- (CIP and FER) Mediterranean. Climatic data collected at four neighboring locations are provided in the Supplementary Material Table S1.

Each of the two bioclimatic zones (Meso- and Supra-Mediterranean) sampled contains different habitats, including (i) densely urbanized habitats (not sampled here), (ii) more or less extensive cultivated areas with berries (*Fragaria* or *Rubus* species) (BAR, CIP, and STJ) or stone fruits (cultivated or wild species of *Prunus*) (BAR, CIP, and STJ), and (iii) natural habitats (BIO, FER, and TIG). Natural habitats obviously differ between the two bioclimatic zones: The main natural vegetation of the Mediterranean Meso is characterized by species such as *Arbustus unedo*, *Cistus sp.*, *Pistacia sp.*, or *Quercus ilex* whereas the Supra-Mediterranean zone is dominated by certain species of *Acer*, *Juniperus communis*, *Pinus sylvestris,* or other *Quercus* species. Potential host plants for *D. suzukii* observed in the close vicinity of the traps (<50 m) are detailed in Table 1. These lists are of course not exhaustive and, among the potential host plants cited by [11,13], several species are widespread both in the Meso- and Supra-Mediterranean: *Cornus sanguinea*, *Hedera helix*, *Sambucus nigra*, *Viburnum lantana*, *Viscum album*, etc. Finally, *Vitis vinifera vinifera* whose fruits can be infested by Ds [18,19,20] is also common as a cultivated, ornamental, or wild plant in the Meso-Mediterranean, especially near BAR and STJ.

### 2.2. Sampling Methods

Two sampling methods were chosen: trap exposure under various experimental conditions (see below) using commercialized frozen fruits (FF) baits and the use of traps filled with an attractive solution (“Vinegar-Wine” (VW)).

The Frozen Fruits method was used during seven trapping sessions (Session 1 to Session 7: end of August 2014; early November 2014; beginning of February 2015, mid-April 2015; end of June 2015; end of August 2015; and end of October 2015) with four simultaneously tested experimental conditions and two replicas per experimental condition for each session and each of the six locations (total of 336 traps).

Commercialized frozen fruits used were a mix of organic blackberries, blackcurrant, raspberries, and cherries provided in standard plastic translucent containers (round boxes, 9 cm basal diameter and height) that had three side holes (diameter: about 1 cm) that could be opened (Conditions 1, 2, and 4) or closed (Condition 3), thus preventing the exclusion of competitors or parasitoids while exposing the larvae to similar abiotic factors. The containers were hooked to the plants (height < 2 m) with wire. We were careful to avoid direct exposure of traps to sunlight during or near zenith hours. To the extent possible, contact with leaves or stems was avoided to prevent or at least to slow colonization by predators (e.g., ants). Frozen fruits were chosen as baits for three reasons: (i) berries and cherries were preferred to other fruits (e.g., bananas) due to their supposed strong attractiveness for Ds and their lower attractiveness for common native *Drosophila* species, (ii) frozen fruits were preferred over fresh fruits based on preliminary observations evidencing a fast rotting process of the latter during field exposure, and (iii) commercialized frozen fruits were the best solution for a consistent supply at an affordable price.

The four tested experimental conditions were:

- Condition 1 = Frozen fruits alone in open traps
- Condition 2 = Frozen fruits previously infested by Ds in open traps
- Condition 3 = Frozen fruits previously infested by Ds in closed traps
- Condition 4 = Frozen fruits previously infested by *D. melanogaster* in open traps.

Infestations by Ds (Condition 2 and Condition 3) or *D. melanogaster* (Condition 4) were performed in large cages using flies maintained under laboratory conditions (21–23 °C, light/dark (L/D):16/8, and relative humidity (RH) 50–70%) for 4 days and 2 days, respectively. Since neither the number of adult flies in the cages nor their physiological state were monitored, ‘laboratory controls’ were systematically carried out for both species, i.e., similar fruit exposures but with a consecutive development in the laboratory. The duration of field exposure was about 7 days for each experimental condition, allowing some flies to develop to the pupal stage. This timing was supposed to also avoid the emergence of adults, although this was probably not always the case during Session 6 (see Supplementary Material Table S1). The traps were returned to the laboratory and maintained at 21–23 °C, L/D:16/8, and RH 50–70% for about one month. All insects emerging from the fruits (*Drosophila* or parasitoids) were collected, counted, and stored in 100% ethyl alcohol for further identification. From Session 3 (February 2015), the protocol was improved to also collect and identify the adult fruit flies present in the cage at the end of the field exposure. It should be noted that the trap closure (Condition 3) proved ineffective during the first session (missing data) but it was subsequently improved.

The “VW” method was used from Session 3 (February 2015) in only three locations (BAR, CIP, TIG) with only one replicate per location. It relies on the use of commercialized red traps (Biobest Droso-traps^®^) filled with home-made bait (2/3 apple cider vinegar, 1/3 red wine). These traps were installed in the same location as the Frozen Fruits traps and removed concomitantly. Living or dead fruit flies in the traps were then collected and stored in 100% ethyl alcohol for further identification.

### 2.3. Species Identification

Most of the fruit flies were first identified morphologically using a “Keys Identification Guideline” for the local *Drosophila* species provided by R. Allemand (Laboratoire de Biologie et Biométrie Evolutive, University Claude Bernard Lyon 1, France). Unidentified individuals were then submitted to J. David for a more accurate determination. Finally, some individuals (including “unidentified” ones) were further identified by sequencing part of the mitochondrial cytochrome oxidase I gene using a routine protocol [21,22,23]. Overall, we were rather confident about most identifications, although, for example, some *D. ambigua* might have been mixed up with *D. subobscura*. The parasitoid species were identified using classical morphological characters. Other arthropods were also present, especially in the “Frozen Fruits” traps, including some potential predators (ants, Vespidae species, or some Staphylinidae). These data are not analyzed here.

### 2.4. Statistical Analysis

All the analyses were performed using the R software (version 3.0.3, R Development Core Team, Vienna, Austria) and related packages, notably “mulcomp” and “Rcmdr”. Multivariate analyses—Factorial Correspondence Analysis (FCA) and Principal Components Analysis (PCA)—were used to investigate the spatiotemporal distribution of the fruit flies (data from “Vinegar—Wine” and from Condition 1 of the “Frozen Fruits” method being treated separately) or the parasitoid species (Condition 1 of the “Frozen Fruits” method). Generalized Linear Models (GLM) were used to test the effect of the different experimental (e.g., Different experimental conditions of the “Frozen Fruit” method) and environmental factors such as Space (“Type of Habitat” and “Location“) and Time (“Sampling Date“) on various aspects of *D. suzukii* biology: presence/absence, survivability, ability to reproduce. For spatial factors, “Location” was nested in “Habitat Type”. The experimental, spatial, and temporal factors were then crossed, and no interaction was considered. Depending on the response variable, Binomial or Poisson distributions were used. For each model, residual diagnostic plots were checked visually. An analysis of the deviance was then carried out, followed by *post hoc* comparisons using the Tukey honest significant difference (HSD) test for significant factors. According to the deviance analysis, a single spatial factor (“habitat type” or “location”) was retained for this purpose.

## 3. Results

### 3.1. Influence of the Trapping Method on the Fruit Flies Sampling

Taken as a whole, the drosophilid species retrieved were (alphabetical order): *Drosophila (Sophophora) ambigua* Pomini 1940, *Drosophila (Dorsilopha) busckii* Coquillet 1901, *Drosophila (Sophophora) helvetica* Burla 1948, *Drosophila (Drosophila) hydei* Sturtevant 1921, *Drosophila (Drosophila) immigrans* Sturtevant 1921, *Drosophila (Drosophila) kuntzei* Duda 1924 (mycophagous fly), *Drosophila (Sophophora) melanogaster* Meigen 1830/*Drosophila (Sophophora) simulans* Sturtevant 1918 (not distinguished), *Drosophila (Drosophila) phalerata* Meigen 1830 (mycophagous fly), *Drosophila (Sophophora) subobscura* Collin 1936, *Drosophila suzukii* Matsumura 1931, *Gitona distigma* Meigen 1830 (a possible predator at the larval stage) and *Hirtodrosophila cameraria* Haliday 1833.

Information on the diversity and abundance of fruit flies was obtained using three complementary approaches (see Materials and Methods): from (i) adult flies (hereafter “wild adults”) found in the traps of the Condition 1 (fruits alone) of the FF method; (ii) flies having emerged from the same traps after their return to the laboratory (hereafter “lab offspring”); and (iii) flies found in the VW traps. Data from these three methods could only be compared for three locations (BAR, CIP, TIG), from February 2015 (Session 3) to October 2015 (Session 7). Overall, the total number of collected individuals largely varied according to the sampling method. Indeed, 2097 individuals were collected with the VW method compared to 453 “wild adults” and 5079 “lab offspring” with the FF sampling. The relative frequencies of the different species also differed depending on the sampling method. With the VW traps, eight drosophilid species were identified: *D. suzukii* (56%), *D. subobscura* (18%), *D. immigrans* (10%), *H. cameraria* (6%), *D. melanogaster*/*D. simulans* (4%), *D. phalerata* (3%), *D. hydei* (1%), and *D. busckii* (less than 1%). All these species were also recovered as “wild adults” with the FF method, but in a very different proportion: *D. melanogaster*/*D. simulans* (89%), *D. suzukii* (6%), *D. subobscura* (4%), and other species (less than 1%). The “lab-offspring” adults (FF method) only belonged to four species: *D. melanogaster*/*D. simulans* (96%), *D. suzukii* (2%), *D. subobscura* (2%), and *H. cameraria* (less than 1%).

### 3.2. Diversity and Abundance of Drosophilids Species

#### 3.2.1. Vinegar—Wine Method (VW)

Of the 2097 individuals collected (all species pooled), 63% came from the BAR location (low elevation, cultivated area); the TIG (low elevation, natural habitat) and CIP (mountainous backland) sites contributed the remaining 21% and 16%, respectively.

Most individuals were collected in mid-April (43% of collected individuals) and at the end of October (28%) while few individuals (3%) were observed in August. The temporal dynamics were characterized by dominance of *D. subobscura* in fruit fly communities early in the year (50% in February, *n* = 202) and of *D. suzukii* later (61% between April and October 2015, *n* = 1895).

**Figure 1:**
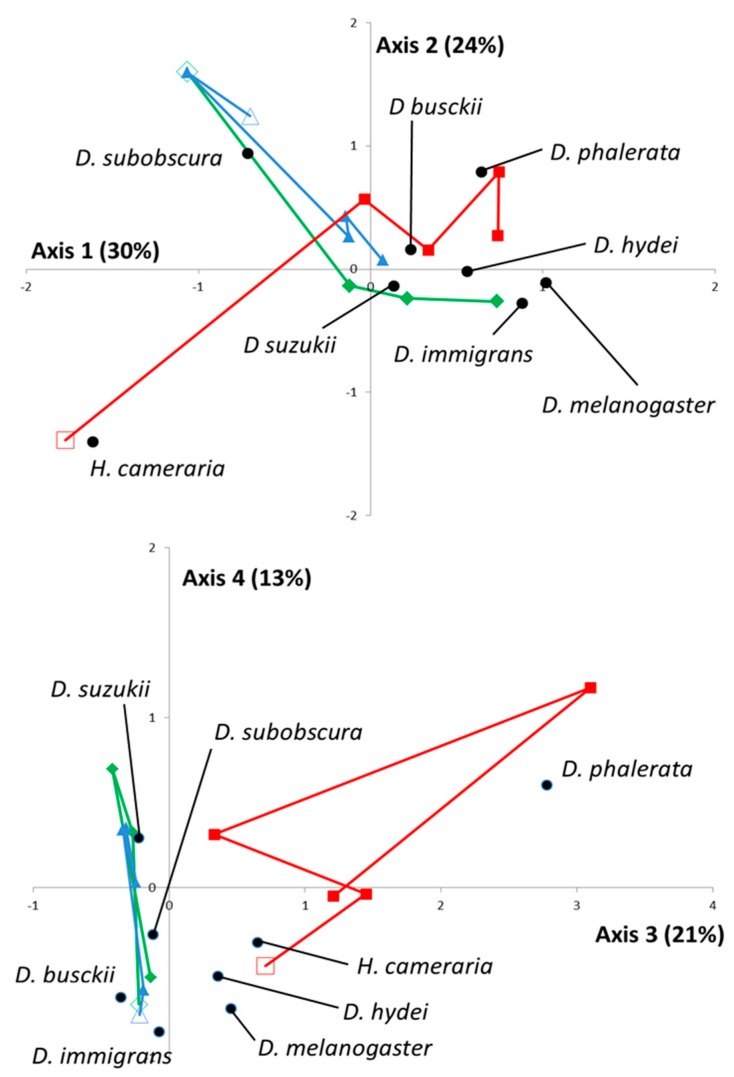
Factorial correspondence analysis (FCA) on wild adults collected with the “Vinegar-Wine” method. The FCA analysis was performed on the table of contingency obtained from the distribution of each of the drosophilid species in each “location *x* date” combination. The projected inertia is provided for each axis. The “location x date” combination is visualized as follows. For the “locations”, green diamonds, red squares, and blue triangles represent BAR (low elevation—cultivated area), TIG (low elevation—natural habitat), and CIP (mountainous backlands), respectively (see also Table 1). For the “dates”, empty figures indicate the starting date (February 2015). The projection of each drosophilid species is explicitly indicated by a black point.

In addition to this global pattern, the FCA was used to highlight spatiotemporal interactions. From a statistical point of view, the first four eigenvalues represented 88% of the total inertia. Sampling data and projections of each *Drosophila* species were, therefore, plotted along the corresponding axis (Figure 1).

Overall, the 1st, 2nd, and 4th axes were useful *a posteriori* for discrimination between Drosophila species, while the 3rd axis clearly evidenced the specificity of the TIG location (natural habitat in Meso-Mediterranean) compared to the other two. Specifically, TIG was indeed characterized by a high proportion (65%, *n* = 115) of *H. cameraria* in February 2015 (Figure 1—upper) and a constant presence of *D. phalerata* (13%, *n* = 57) (Figure 1—lower). The temporal dynamics were rather similar for the two other locations, BAR and CIP (cultivated areas, Meso- and Supra-Mediterranean, respectively; Figure 1—upper), during most of the year but a site-specific enrichment in *D. melanogaster* and *D. immigrans* occurred in BAR in October 2015. Overall, the spatiotemporal variations of *D. suzukii* were rather similar to those observed with *D. busckii* (close projections on the 1st, 2nd, and 3rd axis).

#### 3.2.2. Frozen Fruits Method (FF)—Wild Adults

A total of 904 individuals were analyzed since three of the individuals collected using this method could not be identified. Most of these individuals came from the two cultivated areas at low elevation, BAR (33%) and STJ (24%), followed by the two natural habitats at low elevation (BIO and TIG) with, respectively, 11% and 20%. Finally, individuals collected in the two mountainous locations (CIP and FER) corresponded to only 5% and 6%, respectively. With this method, the number of individuals collected increased slowly but regularly (from 3% to 9% of the collected individuals) between February and August, and then increased quickly in late October (79% of the individuals). The fruit fly community was dominated by *D. suzukii* in February 2015 (85%, *n* = 26), followed by *D. subobscura* in April and June (61%, *n* = 79) and finally by *D. melanogaster*/*D. simulans* (70%, *n* = 796) in August and October. This pattern is, therefore, quite different from that obtained using the VW method.

**Figure 2:**
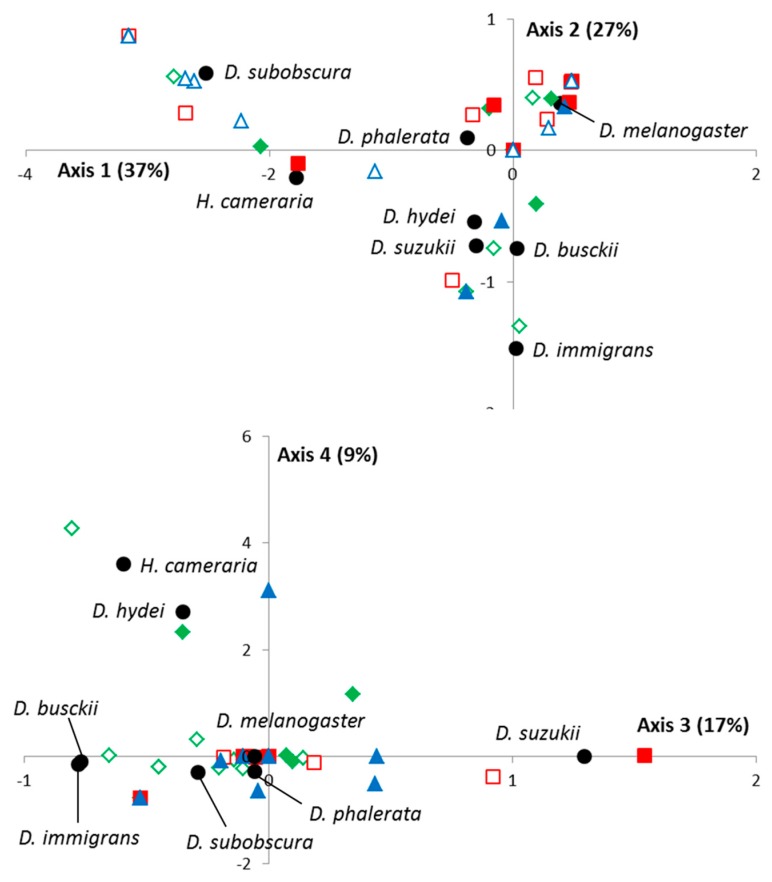
Factorial correspondence analysis (FCA) on wild adults collected in the Condition 1 (fruits alone) of the “Frozen Fruits” method. The FCA analysis was performed on the table of contingency obtained from the distribution of each of the drosophilid species in each “location/replicas *x* date” combination. The projected inertia is provided for each axis. The code for the “locations” and “replicas” is as follows: (i) Green = low elevation—cultivated area (plain signs = BAR; empty signs = STJ); (ii) Red = low elevation—natural habitat (plain signs = TIG; empty signs = BIO); (iii) Blue = mountainous backland (plain signs = CIP; empty signs = FER) (see also Table 1). Unlike Figure 1, no temporal information is provided here (see Results). The projection of each drosophilid species is explicitly indicated by a black point.

As for the VW method, an FCA analysis was performed, with the first four eigenvalues representing 90% of the total inertia. As shown in Figure 2, no spatial segregation was observed, records mapping being explained by differences between drosophilid species, temporal variations, and/or date-by-location interactions. However, the spatio-temporal variations of *D. suzukii* were also close here to those of *D. busckii* (close projections on the 1st, 2nd, and 4th axis) and to a lesser extent *D. hydei* (1st and 2nd axis).

### 3.3. Specific Focus on D. Suzukii (Ds)

#### 3.3.1. Field Reproduction Ability

Ds adult flies were consistently found in collected samples whatever the trapping method (VW, FF “wild adults”, FF “lab offspring”), the sampling location, or the date. The reproductive ability of Ds females was assessed based on the occurrence of Ds offspring in “Condition 1” (frozen fruits alone) and “Condition 4” (frozen fruits previously infested by *D. melanogaster*). A significant difference was observed between collection dates (χ^2^_6df_ = 18.46; *p* = 0.005) with a 27% mean proportion of traps containing Ds offspring (*n* = 140 traps) except in February 2015 (only 4%, *n* = 24 traps). No significant difference was observed between habitats (low-altitude natural habitat, low-altitude cultivated areas, and mountainous backland; GLM model assuming a binomial distribution: χ^2^_2df_ = 1.11; *p* = 0.57) or replicates within habitats (χ^2^_3df_ = 2.07; *p* = 0.56). Ds females were thus able to lay eggs in most spatial and temporal conditions encountered in the sampling area.

#### 3.3.2. Pre-Imaginal Survival

**Figure 3:**
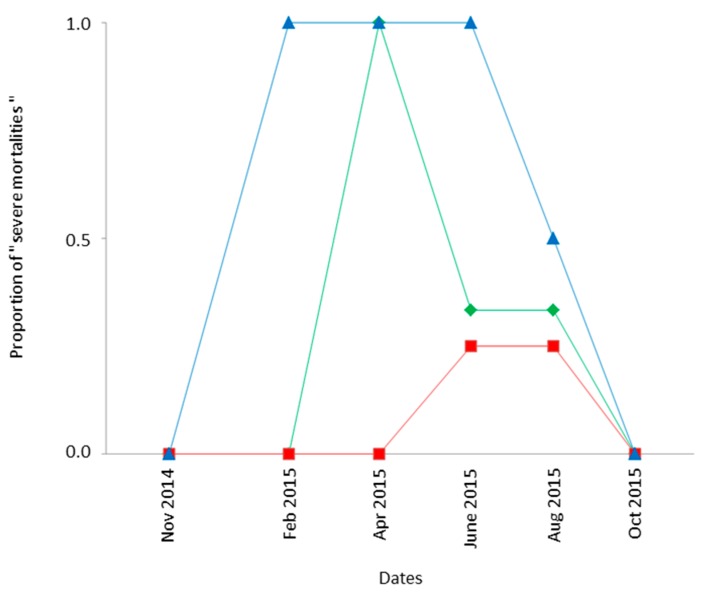
Spatiotemporal distribution of “severe mortality” of pre-imaginal *Drosophila suzukii* (Ds). The term “high mortality” refers to cases where the number of Ds from closed traps exposed in the field (Method 3; “Frozen Fruits” method) represents less than 10% of their number in the corresponding laboratory controls. Based on the analysis of deviance, data from the two “locations” within each “habitat” were pooled. Green diamonds, red squares, and blue triangles, respectively, represent the “low elevation—cultivated” (BAR and STJ), “low elevation—natural habitat” (BIO and TIG), and “mountainous backlands” (CIP and FER) areas (see also Table 1).

Spatiotemporal variations in survival of field-exposed Ds eggs/larvae were assessed by comparing the “Condition 3” (frozen fruits previously infested by Ds in closed traps) and “Laboratory controls” (similarly infested fruits kept in the laboratory). Except for 2 out of 60 cases, the number of Ds adults recovered after field exposure was lower than in controls: the median percentage of successful development outdoor was only 35% of that in the laboratory. The percentage was even below 10% of that of controls in 30% of cases, such events corresponding to “severe field mortalities”. A detailed analysis of these severe field mortalities evidenced a significant influence of both the habitat and the date (GLM model assuming a binomial distribution: χ^2^_2df_ = 14.24; *p* < 10^−3^; χ^2^_5df_ = 27.96; *p* < 10^−3^, respectively) but not of the location within a given habitat (χ^2^_3df_ = 4.47; *p* = 0.21). As shown in Figure 3, severe field mortality of field-exposed Ds occurred more often in mountainous locations (60% of cases; CIP and FER) compared to cultivated areas (BAR and STJ, 24% of cases) and low-elevation natural habitats (BIO and TIG; 9% of cases). It nevertheless occurred more frequently (mean of 48% of cases) in spring and summer (April–September 2015) than in other seasons, including winter.

#### 3.3.3. Offspring Production and Distribution

The number of Ds that emerged from the traps is the result of several sequential factors or steps including: (i) the local presence of adult females; (ii) their ability to find the traps and their decision to enter them; (iii) their ability to lay eggs; and (iv) the ability of larvae to develop. Overall, Ds offspring was recovered in 24% of the FF traps from the “Condition 1” (frozen fruits alone) and “Condition 4” (frozen fruits previously infested by *D. melanogaster* (Dm)). The number of Ds per trap varied between 0 and 87, with no individual in 76% of the traps and more than 10 individuals in only 5% of the traps. Such a distribution prevented any quantitative approach. The effects of the environmental (space: habitat and location—time: sampling date) and the experimental (previous infestation by Dm or not) factors were thus qualitatively investigated using a GLM on the presence/absence of Ds offspring (binomial distribution) (Table 2—top). Among the environmental factors (space and time), only the “Date” factor was significant, Ds being observed in less than 10% of the traps at the beginning of February 2015 and the end of October 2015. The experimental factor (previous infestation by Dm or not) was also not significant.

The reciprocal situation—the influence of the previous infestation by Ds on the presence/absence of Dm offspring—was also investigated. As shown in Table 2 (lower), the distribution of Dm was strongly influenced by environmental (space and time) but not by experimental (previous infestation by Ds) factors.

On the whole, these results do not evidence interactions between *D. suzukii* and *D. melanogaster*, be they negative (repulsion and/or competition) or positive (attraction). It should be noted, however, that the amount of food was probably not limiting.

### 3.4. Occurrence of Parasitoids

#### 3.4.1. Diversity, Abundance, and Spatiotemporal Correlations for Parasitoids

No new species were observed compared to previous investigations in the south of France [24]. The individuals belonged to one of the four following species: *Leptopilina boulardi* Barbotin, Carton and Keiner-Pillaut 1979, *Leptopilina heterotoma* Thomson 1862 (Hymenoptera: Figitidae), *Pachycrepoideus vindemmiae* Rondani 1875 (Hymenoptera: Pteromalidae), and *Trichopria cf drosophilae* (Hymenoptera: Diapriidae).

**Figure 4:**
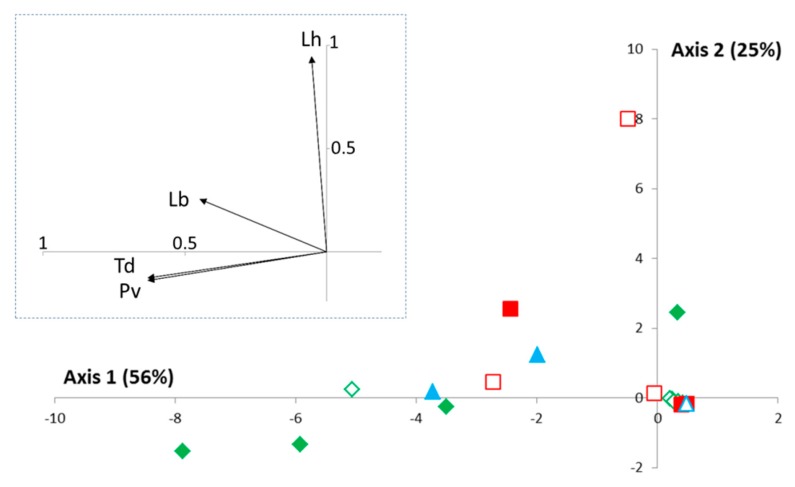
Principal components analysis (PCA) on parasitoids collected in Condition 1 (fruits alone) of the “Frozen Fruits” method. The PCA analysis was performed from the distribution of the different parasitoid species in each of the “location/replicas *x* date” combinations. The associated inertia is provided for each axis. The code for the “location” is as follows (see also Table 1): (i) Green = low elevation—cultivated area (plain signs = BAR; empty signs = STJ); (ii) Red = low elevation—natural habitat (plain signs = TIG; empty signs = BIO); (iii) Blue = mountainous backland (plain signs = CIP; empty signs = FER). Contrary to Figure 1, the temporal dynamics were not explicitly shown here (see Results). The correlations between each PCA axis and each parasitoid species are presented in the grey square (rescaled and shifted for a better readability). Lb, Lh, Pv, and Td correspond to *Leptopilina boulardi*, *L. heterotoma*, *Pachycrepoideus vindemmiae,* and *Trichopria cf drosophilae*, respectively.

In the survey of 244 traps, a total of 3813 parasitoids emerged from “Condition 1” (frozen fruits alone), “Condition 2” (frozen fruits previously infested by *D. suzukii*), and “Condition 4” (frozen fruits previously infested by *D. melanogaster*). The four parasitoids species identified were *Leptopilina boulardi* (47% of individuals; 15% of traps), *Leptopilina heterotoma* (less than 1% of individuals; 2% of traps), *Pachycrepoideus vindemmiae* (31% of individuals; 16% of traps), *Trichopria cf drosophilae* (21% of individuals; 16% of traps). In the principal components analysis performed using only traps of “Condition 1” (frozen fruits alone), 81% of the variability was explained by the two first axes (Figure 4), with a strong correlation between the three main species and the first axis. Similar spatiotemporal patterns were notably observed for *P. vindemmiae* and *T. cf drosophilae*. The second axis mainly explained the distribution of the rare species *L. heterotoma* for which 3 of the 4 individuals were found together (BIO in August 2014). Of course, the small number of individuals precludes further ecological interpretations.

#### 3.4.2. Comparison between Non-Infested and Previously Infested Traps

The influence of the “trapping method” and the environmental factors (geography and date) on the occurrence of parasitoids was tested only for the three most abundant and widespread species (*L. boulardi*, *P. vindemmiae*, *T. cf drosophilae*) (Table 3). It is noteworthy that none of the species were observed in mid-April 2015 (Session 4).

For *L. boulardi* (Lb), all tested factors were significant. This species was found less frequently in traps previously infested by *D. suzukii* (Condition 2—9% of traps) than in those that were non-infested (Condition 1—19% of traps) (Tukey HSD: *z* = 2.34 with *p* = 0.05) or *D. melanogaster*-infested traps (Condition 4—17% of traps) (*z* = 2.67 with *p* = 0.02). Lb were also observed at a high frequency in November 2014 (64% of traps) and October 2015 (28% of traps) compared to at other dates (mean occurrence in 2% of traps—see Supplementary Material Table S2 for pairwise comparisons). Finally, Lb occurred in 32% of the traps in STJ and about 14% in BAR, BIO, TIG, and CIP, whereas it was never observed in FER, the most mountainous and less anthropized location.

For *P. vindemmiae* (Pv), the trap condition, the sampling date, and the habitat were significant. The species occurred more frequently in traps previously infested by *D. melanogaster* (Condition 4—20% of traps) than in non-infested ones (Condition 1—11% of traps) (Tukey HSD: *z* = 2.34 with *p* = 0.05). Although the same trend was observed for *D. suzukii*-infested traps (Condition 2—21% of traps), the difference was not significant (*z* = 1.94 with *p* = 0.13), possibly due to a lower sample size. The same was observed for Condition 2 and Condition 4 that were not significantly different (*z* = 0.36 with *p* = 0.93). Pv was more frequently observed in November 2014 (67% of traps) than in all other sessions (mean occurrence in 9% of traps—see Supplementary Material Table S2 for pairwise comparisons). It also occurred more frequently in cultivated areas (BAR and STJ—mean occurrence in 29% of traps) than in wild habitats at low elevation (BIO and TIG—mean occurrence in 13% of traps) (*z* = 2.86 with *p* = 0.01) or in mountainous backland (CIP and FER*—*mean occurrence in 9% of traps) (*z* = 3.62 with *p* < 10^−3^). The difference between these two last habitats was not significant (*z* = 1.15 with *p* = 0.48).

The results for *T. cf drosophilae* (Td) were roughly similar to those for Pv, with three significant variables: the trap condition, the sampling date, and the habitat. Td was more frequently observed in traps previously infested by *D. melanogaster* (Condition 4—21% of traps) or *D. suzukii* (Condition 2—22% of traps) than in non-infested traps (Condition 1—11% of traps), but the difference was only significant in the first case (Condition 4 versus Condition 1: *z* = 2.50 with *p* = 0.03—Condition 2 versus Condition 1: *z* = 2.08 with *p* = 0.09), likely because of a lower sample size. The difference between Condition 2 and Condition 4 was also not significant (*z* = 0.39 with *p* = 0.92). Td was also more frequently observed in November 2014 (presence in 67% of the traps) compared to all other dates (mean occurrence in 10% of traps—see Supplementary Material Table S2 for pairwise comparisons). Finally, Td seems to occur more frequently in cultivated areas (27% of traps) than in wild habitats at low elevation (17% of traps—*z* = 1.73 with *p* = 0.19) or in mountainous backland (10% of the traps—*z* = 3.04 with *p* = 0.007). The difference between the two last habitats was not significant (*z* = 1.54 with *p* = 0.27).

## 4. Discussion

This survey was performed in the frame of the assessment of classical biological control against *D. suzukii* (Ds) using parasitoids. More precisely, the aim was to document the occurrence and biology of Ds, as well as ecologically related drosophilids and parasitoids, in a localized area. In addition to being close to our laboratory, the study area was selected on the basis of its climatic, geographical, and ecological heterogeneity, so that these results may be relevant, to a certain extent, for a much wider geographical perimeter: similar Mediterranean regions in France (Provence Alpes Côte d’Azur, Languedoc-Roussillon, and South Rhône-Alpes) and European countries such as Italy (Piemont, Liguria, and north of Toscana) or Spain (Catalonia).

The first lesson to be learned from this field work is a methodological warning insofar as, for example, the relative frequencies of *Drosophila* species varies considerably according to the sampling method (FF versus VW methods). For example, Ds could be considered the most abundant species or a rather rare species according to the sampling method. It also determines whether the presence of certain species (e.g., *D. busckii*, *D. hydei,* and *D. phalerata*) will be detected or not. Such a discrepancy is not a surprise *per se* and can mainly be explained by interspecific differences in olfactory cues used for egg laying or adult nutrition, on the one hand, and the suitability of the rearing media for pre-imaginal development, on the other hand (see for instance [25,26,27]). However, this is a challenge when the objectives are to quantify Ds populations or to picture the drosophilids community as desired or requested in pre- or post-release surveys related to the introductions of exotic biological control agents. Of course, our own experimental design may also suffer from some biases such as (i) the presence of detrimental chemical or natural products in the frozen fruits, (ii) intra or inter-trap variations in exposure to sunlight, (iii) possible greenhouse effect in traps, and (iv) a variable (spatial and/or temporal) “attraction” of our own traps based on available resources for *D. suzukii*. To improve our knowledge of the pre-release state, complementary experiments are thus underway on the actual spatiotemporal use of some frequent cultivated/wild fruits by Ds and other related species (drosophilids and parasitoids).

A second interesting result is the spatio-temporal co-occurrences between Ds, *D. busckii,* and *D. hydei*, as these three species apparently share common biological requirements (Figure 1 and Figure 2). To date, we do not know if these co-occurrences lead to interspecific competition or other types of interactions. However, as part of the investigations into the ecological impacts of *D. suzukii* in the invaded areas, such a result clearly suggests it will be necessary to pay more attention to these species that have been neglected with regard to *D. melanogaster* [20,28,29]. Indeed, even if Ds does interact with *D. melanogaster*/*D. simulans* (see for instance the “vineyard” case-study: [18,19,20]), it seems that most of the regular resources for *D. melanogaster*/*D. simulans*, whether natural (e.g., fallen fruits) or artificially introduced (e.g., banana-filled traps), are probably not the most suitable for this species. For instance, a survey carried out on a French scale (10 sites between 43.5° and 45° N latitude) failed to detect Ds in banana traps in most of the cases, although the pest was present in neighboring crops ([30], unpublished observation). Additionally, the identification of native *Drosophila* species closely ecologically related to Ds is of first interest in the evaluation of exotic biological control agents.

The third contribution of the study is in regards to the ecology of Ds in the study area. Despite the contrasted climatic and ecological conditions, Ds flies seem to be present at the pre-imaginal and/or adult stages in all the spatiotemporal conditions. Besides, the most severe mortalities at the pre-imaginal stage were not necessarily observed in the coldest conditions (mountainous backland in December-February) but later in spring. Therefore, and despite possible biases, this suggests that the highest pre-imaginal mortality may not be due to the low temperatures but to other abiotic factors [31,32]. As a result, adults are probably produced continuously in the local area, females being also able to reproduce for most of the year, and more attention needs to be given to the spatial and temporal occurrence of reproductive diapause [33,34]. In several respects, our results confirmed those obtained in the Italian Alps [35] and the low control of Ds by local climatic conditions.

Finally, this study provides insights about the possibility of recruitment of native parasitoids using a bait (frozen berries) more favorable to Ds with regard to other competitors. As observed elsewhere with other methods (Europe: [36,37,38,39]—other continents: [39,40,41,42]), the two pupal parasitoids *P. vindemmiae* and *T. cf drosophilae* were found to be the most frequent species, although the methodology used may have been disadvantageous for their sampling (as pupae were not available during the first days of field exposure). These two species are known to be generalists and *P. vindemmiae* can also behave as a hyperparasitoid [43,44]. However, their abundance in our traps was rather limited to the late fall, and we found little evidence of regulation by larval parasitoids. *L. heterotoma* was previously described as the most generalist larval parasitoid based on laboratory experiments (see for instance [13] and references herein), and thus the most likely to behaviorally or physiologically adapt to Ds. Such adaptations have not been observed yet in the field. This species appeared to be very rare during this survey, probably due to competition with *L. boulardi* and/or change in climatic conditions [45]. Not surprisingly, we observed no evidence of Ds parasitism by *L. boulardi*, a specialist of *D. melanogaster* and *D. simulans* (see for instance [13] and references herein). Altogether, data thus suggest that there is room for the introduction of an exotic parasitoid specialized on *D. suzukii* in the south of France and in the neighboring territories. The other two key characteristics for effective or at least significant control would be both the ability to grow early in the year and to successfully locate the target host in its particular ecological niche: the wide variety of cultivated and wild mature fruits.

## Figures and Tables

**Table 1:** Description of the six trapping locations including geographic, climatic, and ecological information, Information on the four climatic references (Cannes, Carros, Caussols, and Saint Cezaire) are provided in the Supplementary Material Table S1. Potential host plants of *D. suzukii* have been defined according to [11,13]. Type of plants: BAR = La Baronne; BIO = Biot; CIP = Cipières; FER = Col Ferrier; TIG = Tignet; STJ = Saint Jeannet; C = Cultivated; W = Wild.

Location	BAR	BIO	CIP	FER	TIG	STJ
Longitude (E)	07.180782	07.077744	6.953913	6.864210	6.833692	7.161173
Latitude (N)	43.722515	43.641776	43.779930	43.712433	43.620803	43.753222
Elevation (m)	150	120	700	1100	150	450
Habitat	Low Elevation Cultivated	Low Elevation Natural	Mountainous Backland	Mountainous Backland	Low Elevation Natural	Low Elevation Cultivated
Climate Reference	Meso Carros	Meso Cannes	Supra Caussols	Supra Caussols	Meso Saint Cezaire	Meso Carros
Sampling Methods	Frozen Fruits Attract. Traps	Frozen Fruits	Frozen Fruits Attract. Traps	Frozen Fruits	Frozen Fruits Attract. Traps	Frozen Fruits
Potential Hosts						
*Arbutus unedo*	-	W	-	-	W	-
*Crataegus monogyna*	-	-	W	W	-	-
*Ficus carica*	W	-	W	-	-	C
*Fragaria vesca*	-	-	-	W	-	-
*Fragaria sp.* (others)	C	-	C	-	-	C
*Prunus armeniaca*	-	-	-	-	-	C
*Prunus avium*	-	-	-	W	-	-
*Prunus domestica*	-	-	-	-	-	-
*Prunus persica*	-	-	-	-	-	C
*Prunus spinosa*	-	W	-	-	W	-
*Prunus sp.* (others)	-	-	-	-	W	-
*Rosa canina*	-	-	W	W	-	-
*Rubus fructicosus*	W	W	W	W	W	W
*Rubus sp.* (others)	-	-	-	W	-	-

**Table 2:** Influence of experimental (Experimental Condition) and environmental (Habitat, Location, and Date) factors on the number of *D. suzukii* (Ds) and *D. melanogaster* (Dm) emerged from the Frozen Fruits traps. Levels of statistical significance: ns: not significant, * : 0.01 < *p*-Value ≤ 0.05; ** : 0.001 < *p*-Value ≤ 0.01; *** : *p*-Value ≤ 0.001.

	Χ^2^	df	*p*-Value	
**Ds Offspring**				
Experimental Condition	1.49	1	*p* = 0.22	ns
Habitat	0.84	2	*p* = 0.66	ns
Location Nested in Habitat	1.82	3	*p* = 0.61	ns
Date	14.81	6	*p* = 0.02	*
**Dm Offspring**				
Experimental Condition	0.91	1	*p* = 0.34	ns
Habitat	9.46	2	*p* = 0.009	**
Location Nested in Habitat	8.46	3	*p* = 0.04	*
Date	82.750	6	*p* < 10^−3^	***

**Table 3:** Influence of experimental (Experimental Condition) and environmental (Habitat, Location, and Date) factors on the presence of three parasitoid species having emerged in Frozen Fruit traps. Levels of statistical significance: ns : not significant, * : 0.01 < *p*-Value ≤ 0.05; ** : 0.001 < *p*-Value ≤ 0.01; *** : *p*-Value ≤ 0.001.

	Χ^2^	df	*p*-Value	
***Leptopilina boulardi***				
Experimental Condition	12.77	2	0.002	**
Habitat	40.18	2	*p* < 10^−3^	***
Location Nested in Habitat	17.14	3	*p* < 10^−3^	***
Date	117.61	6	*p* < 10^−3^	***
***Pachycrepoideus vindemmiae***				
Experimental Condition	6.80	2	0.03	*
Habitat	19.48	2	*p* < 10^−3^	***
Location Nested in Habitat	0.75	3	0.86	ns
Date	67.36	6	*p* < 10^−3^	***
***Trichopria cf drosophilae***				
Experimental Condition	7.75	2	0.02	*
Habitat	11.09	2	0.003	**
Location Nested in Habitat	0.93	3	0.82	ns
Date	65.11	6	*p* < 10^−3^	***

## Supplementary Materials

The following are available online at www.mdpi.com/2075-4450/8/4/124/s1, Figure S1: Localization of the sampling sites on the satellite map. Sampling sites are indicated by color dots on the satellite picture of the Alpes-Maritimes area (from the geoportal, IGN) visualizing their geographical position (Valley, mountainous backlands and distance to the seaside). The large yellowish dot indicates the town of Nice. The position of the Alpes-Maritimes on the France map is indicated in the insert. Table S1: Climatic data related to the investigated area. These four references were chosen for their vicinity to one or two of the sampled locations (see Table 1). MTmin/MTmea/MTmax respectively indicate the monthly means of daily minimal/mean/maximal temperatures while MRH indicates the monthly Mean Relative humidity. Sampling dates are indicated in the “Session” column. Table S2: Summary of post hoc tests (Tukey HSD tests) dealing with the temporal abundances of the three most abundant parasitoids. When the comparison between two sampling dates (S1 to S7—see Material and Methods) was possible, we indicate the observed statistics (above) and the related *p*-value (below).
