# Supplementary material for: Field Monitoring of Drosophila suzukii and Associated Communities in South Eastern France as a Pre-Requisite for Classical Biological Control
Source: Insects. 2017 Nov 16;8(4):124. doi: 10.3390/insects8040124 (PMC5746807; doi:10.3390/insects8040124)
Supplement: Supplementary file 1 [file insects-08-00124-s001.pdf]

insects

Supplementary Materials: Field Monitoring of *Drosophila suzukii* and Associated Communities in South Eastern France as a Pre-Requisite for Classical Biological Control

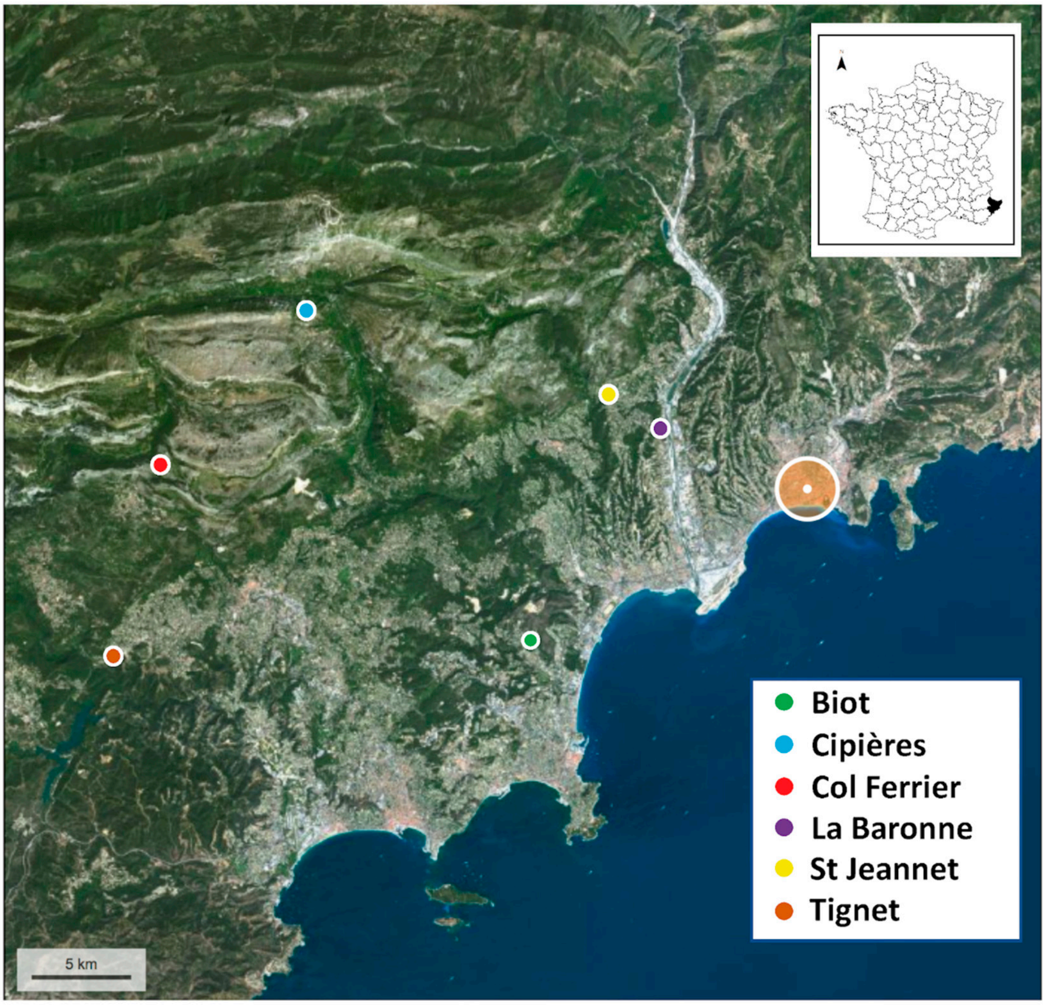

Figure S1. Localization of the sampling sites on the satellite map. Sampling sites are indicated by color dots on the satellite picture of the Alpes-Maritimes area (from the geoportal, IGN) visualizing their geographical position (Valley, mountainous backlands and distance to the seaside). The large yellowish dot indicates the town of Nice. The position of the Alpes-Maritimes on the France map is indicated in the insert.

Table S1. Climatic data related to the investigated area. These four references were chosen for their vicinity to one or two of the sampled locations (see Table 1). MTmin/MTmea/MTmax respectively indicate the monthly means of daily minimal / mean / maximal temperatures while MRH indicates the monthly Mean Relative humidity. Sampling dates are indicated in the "Session" column.

		CANNES				CARROS				SAINT CEZAIRE				CAUSSOLS			
		43.555 N; 6.950 E				43.785 N; 7.208 E				43.678 N; 6.808 E				43.752 N; 6.922 E			
**Date**	**Session**	**MTmin**	**MTmea**	**MTmax**	**MRH**	**MTmin**	**MTmea**	**MTmax**	**MRH**	**MTmin**	**MTmea**	**MTmax**	**MRH**	**MTmin**	**MTmea**	**MTmax**	**MRH**
July 2014		17.3	22.3	27.3	68	17.8	23.4	29.0	_	14.4	19.7	25.0	65	12.7	16.3	19.9	72
August 2014	S1	18.0	23.3	28.7	68	17.7	23.3	28.9	_	14.6	20.4	26.3	64	12.9	16.9	20.9	67
September 2014		16.5	21.4	26.3	72	16.9	22.3	27.7	_	13.6	18.7	23.8	68	11.4	14.7	18.1	74
October 2014		13.7	18.7	23.8	75	14.0	19.0	24.0	_	11.0	15.6	20.2	73	8.8	12.1	15.4	77
November 2014	S2	10.7	14.5	18.4	81	10.3	13.9	17.5	_	7.9	11.1	14.4	80	4.9	7.4	9.8	84
December 2014		5.8	10.6	15.3	77	6.3	9.9	13.5	_	3.7	7.4	11.1	69	1.6	4.5	7.3	65
January 2015		3.1	8.9	14.7	72	3.8	8.8	13.8	_	2.5	6.9	11.3	59	0.2	3.6	7.0	59
February 2015	S3	3.8	8.7	13.7	73	4.2	9.0	13.7	_	1.5	5.6	9.7	67	-1.3	1.9	5.1	69
March 2015		6.5	11.8	17.1	69	7.0	12.3	17.5	_	5.0	9.4	13.9	61	2.5	5.6	8.7	66
April 2015	S4	9.0	13.9	18.8	74	9.2	14.7	20.2	_	7.1	12.1	17.0	67	5.2	8.7	12.1	67
May 2015		13.1	18.2	23.2	70	13.3	18.8	24.2	_	10.9	16.1	21.3	65	9.4	13.2	17.0	63
June 2015	S5	17.0	22.1	27.3	68	17.6	23.4	29.2	_	14.6	19.9	25.2	64	12.6	16.2	19.8	69
July 2015		21.0	**26.2**	31.3	65	21.3	**27.5**	33.7	_	18.6	**24.5**	30.4	58	17.7	**21.4**	25.1	59
August 2015	S6	18.5	23.9	29.3	65	19.9	25.4	31.0	_	16.6	22.0	27.4	59	14.4	18.0	21.6	66
September 2015		15.0	20.4	25.8	64	15.6	21.1	26.6	_	12.4	17.5	22.6	61	9.9	13.5	17.0	67
October 2015	S7	11.5	16.0	20.5	79	11.9	16.5	21.1	_	8.6	12.8	17.0	78	6.6	9.8	13.0	77

Table S2. Summary of post hoc tests (Tukey HSD tests) dealing with the temporal abundances of the three most abundant parasitoids. When the comparison between two sampling dates (S1 to S7—see Material and Methods) was possible, we indicate the observed statistics (above) and the related *p*-value (below).

A—*L. boulardi*						
	S2<br>November 2014	S3<br>February 2015	S4<br>April 2015	S5<br>June 2015	S6<br>August 2015	S7<br>October 2015
S1 August 2014	**−4.50**<br>**$p < 10^{-3}$**	–	–	–	**−4.09**<br>**$p < 10^{-3}$**	**−3.46**<br>**$p = 0.005$**
S2		–	–	–	1.48<br>$p = 0.67$	**3.18**<br>**$p = 0.01$**
S3			–	–	–	–
S4				–	–	–
S5					–	–
S6						2.41<br>$p = 0.12$
B—*P. vindemmiae*						
	S2	S3	S4	S5	S6	S7
S1	**−4.52**<br>**$p < 10^{-3}$**	–	**−4.63**<br>**$p < 10^{-3}$**	**−4.51**<br>**$p < 10^{-3}$**	−4.35<br>**$p < 10^{-3}$**	−4.63<br>**$p < 10^{-3}$**
S2		–	−0.05<br>$p = 1$	0.37<br>$p = 1$	0.74<br>$p = 0.99$	−0.03<br>$p = 1$
S3			–	–	–	–
S4				0.42<br>$p = 1$	0.80<br>$p = 0.98$	0.02<br>$p = 1$
S5					0.38<br>$p = 1$	−0.41<br>$p = 1$
S6						−0.78<br>$p = 0.98$
C—*T. cf drosophilae*						
	S2	S3	S4	S5	S6	S7
S1	**−4.18**<br>**$p < 10^{-3}$**	–	**−4.59**<br>**$p < 10^{-3}$**	**−4.33**<br>**$p < 10^{-3}$**	**−4.33**<br>**$p < 10^{-3}$**	**−4.59**<br>**$p < 10^{-3}$**
S2		–	−1.26<br>$p = 0.84$	−0.08<br>$p = 1$	−0.08<br>$p = 1$	−0.83<br>$p = 0.98$
S3			–	–	–	–
S4				1.20<br>$p = 0.87$	1.20<br>$p = 0.87$	0.49<br>$p = 1.00$
S5					0.00<br>$p = 1$	−0.76<br>$p = 0.99$
S6						−0.76<br>$p = 0.99$

© 2017 by the authors. Submitted for possible open access publication under the terms and conditions of the Creative Commons Attribution (CC BY) license (http://creativecommons.org/licenses/by/4.0/).
